# Risk and Protective Factors Associated With Health-Related Quality of Life of Parents With Mental Illness

**DOI:** 10.3389/fpsyt.2021.779391

**Published:** 2021-12-01

**Authors:** Alina Radicke, Marlit Sell, Bonnie Adema, Anne Daubmann, Reinhold Kilian, Mareike Busmann, Sibylle M. Winter, Martin Lambert, Karl Wegscheider, Silke Wiegand-Grefe

**Affiliations:** ^1^Department of Child and Adolescent Psychiatry, Psychotherapy and Psychosomatics, University Medical Center Hamburg-Eppendorf, Hamburg, Germany; ^2^Department of Medical Biometry and Epidemiology, University Medical Center Hamburg-Eppendorf, Hamburg, Germany; ^3^Department of Psychiatry II, Ulm University at Bezirkskrankenhaus Guenzburg, Guenzburg, Germany; ^4^Department of Child and Adolescent Psychiatry, Psychosomatics and Psychotherapy, Berlin Institute of Health (BIH) and Charité Medical University of Berlin, Corporate Member of Freie Universität Berlin, Humboldt-Universität zu Berlin, Berlin, Germany; ^5^Department of Adult Psychiatry, Psychotherapy and Psychosomatics, University Medical Center Hamburg-Eppendorf, Hamburg, Germany

**Keywords:** health-related quality of life, parents with mental illness, psychopathology, family psychology, family burden, parental mental disorder

## Abstract

**Purpose:** Health-related quality of life (HRQoL) can be reduced in parents with mental illness (mental illness) who face the dual demands of disabling symptoms and their impact on family, social, and occupational life. This study aimed at analyzing the influence of various factors on HRQoL in parents with mental illness.

**Method:** Baseline data of the German randomized controlled multicenter project CHIMPS (children of parents with mental illness) was used for analyses. The final sample consisted of *n* = 208 parents with mental illness and *n* = 197 children and adolescents aged 8–18 years. HRQoL was assessed with the EQ-5D.

**Results:** Parents with mental illness reported significantly lower global and specific HRQoL than the German reference population. They were least satisfied with aspects that relate to anxiety and depression followed by usual activities, pain and discomfort. Better global HRQoL was primarily associated with self-reported physical and mental health, as well as adaptive coping behavior. Associations with mobility, self-care, usual activity, pain and discomfort, anxiety and depression were analyzed and discussed.

**Conclusions:** HRQoL in parents with mental illness is reduced. Clinical interventions should focus on the alleviation of mental health symptoms and probably somatic symptoms and promote adaptive coping skills.

## Introduction

Worldwide, about 15–23% of families have or had to deal with mental health symptoms in at least one parent (1, 2). Parents with mental illness may struggle to deal with the dual demands of disabling symptoms and their impact on family, social, and occupational life. Mothers with mental illness have reported severe financial strains and high unemployment rates, poor physical health, many stressful life events, and a high prevalence of custody loss (2–5). In addition, parents face practical challenges that require time and energy, for example, obtaining necessary services and supports for themselves and their family, and implementing treatment regimens (5, 6). Parents with mental illness may also have to deal with psychological difficulties in their children who have an increased risk to develop mental health symptoms compared to the general population due to various genetic and environmental factors (2, 7–9). Taken together, these challenges constitute a risk factor for the quality of life in parents with mental illness.

Research has consistently shown that quality of life is reduced in adults with mental illness (10–12). In combination with the demands of parenthood, adults with mental health symptoms may face even more stressful situations that lower their health-related quality of life (HRQoL). On the other hand, parents who experience personal growth through childrearing (13) may have improved HRQoL. HRQoL has become an outcome criterion to evaluate current functioning, the burden of mental illness, as well as treatment effectiveness (14). In recent years, the treatment goals for people with mental illness shifted from the pure alleviation of mental health symptoms to the improvement of the patient's satisfaction with quality of life and social activities (15). It has been suggested that symptom reductions do not solely improve quality of life, because other symptoms remain (e.g. difficulty with everyday functioning, financial strains, stigmatization, loneliness) (16). It is crucial to identify modifiable risk and protective factors linked to the parents' HRQoL that can be addressed for psychological interventions and support programs to ultimately improve the patients' HRQoL. Because the patient's HRQoL and mental health is linked to the quality of life of the people surrounding the person including children and spouses improving HRQoL in parents with mental illness may also have additional benefits for their family's life satisfaction (8, 17, 18).

Various characteristics of mental illness influence the HRQoL outcome for mental illness. For example, a recent meta-analysis (19) summarized that reduced quality of life in people with psychotic disorders is associated with the higher symptom severity and a longer duration of untreated psychosis. Quality of life in people with mental illness is also influenced by premorbid adjustment (20, 21), comorbid mental diagnoses (16, 22, 23). Coping behavior, which aims at the reduction and management of stressful experiences that exceed one's resources (24), has an important impact on HRQoL (25) and it predicts a broad range of health outcomes over time (26, 27). Parents can respond to stress in adaptive and maladaptive ways. Maladaptive coping includes avoidant and emotion-focused strategies (e.g. denial, self-blame, substance use) that are considered to be risk factors for poor mental health (28–30) and HRQoL (25). Adaptive coping promotes good mental health (31–33) and includes active problem-oriented strategies like making a plan of action to deal with mental illness and its consequences, and actively exchange experiences with peers, as well as emphasizing one's strengths and positive attributes (30). Parents who appraise and cope with their mental illness in an adaptive way, show less symptom severity over time, reduce the symptom burden as well as comorbid mental health symptoms, and have a shorter illness duration (34). Adaptive coping can also reduce the family burden and stigmatization for their mental health condition (35).

Family relationships can either provide social support and buffer against stress or they can cause distress and diminish the HRQoL of parents with mental illness (36). Several studies have shown that family functioning is reduced in people with various mental disorders (36–41). Family dysfunction associated with parents' mental illness is associated with family discord, lower levels of expressiveness and affective involvement, and impaired communication (36–39). The mental disorders' impact on family functioning has been partially determined by illness characteristics including symptom type and severity, a relapse frequency and the severity of impaired functioning (42). Adverse psychosocial outcomes (e.g. unemployment, poverty, loss of custody) and the presence of psychological difficulties in their children can further strain the family's relationships (2, 6, 43). When children of parents with mental illness suffer from mental health symptoms, parents experience additional distress and diminished quality of life (44, 45). They may worry about the impact of their mental illness on their children, feel guilty and may require additional social support (46). Social support from friends and sources outside the family are important for the mental health and quality of life of parents with mental illness (47–49). For example, a randomized controlled trial found that patients with psychosis who participated in peer support groups reported a greater increase in self-efficacy, social support and quality of life than did the control group (47). Similarly, the predecessor CHIMPS study, a pre-post pilot trial involving a waiting-list control group showed that the manualized CHIMPS intervention improved children's social support mental health and quality of life along with family relationships and parents' coping strategies (50–54). About one third of parents with mental illness perceive their social support as insufficient (52). They are less satisfied than welfare recipients and people of the general populations with the availability and adequacy of social support, particularly with regard to child rearing indicating a need to improve the availability of social support with adequate intervention (49).

The purpose of the present study is to identify risk and protective factors associated with HRQoL in parents with mental illness. This study extends previous research by considering HRQoL aspects that are particularly relevant for parents with mental illness (e.g., child mental illness, social support). Moreover, research on the utility of generic measures like the EQ-5D in populations with mental illness is still limited. Testing the appropriateness of generic measures like the EQ-5D in mental health populations enables clinicians and economics to gain a more realistic picture of the respondents' mental health and the (cost-) effectiveness of clinical interventions.

## Materials and Methods

### Study Design

We analyzed baseline data of the German “CHIMPS” (children of parents with mental illness) project collected between 2014 to 2017. The randomized controlled multicenter study “Implementation and evaluation of a family-based intervention program for children and adolescents of parents with mental illness” addressed parents with mental illness, their children, and partners, and aimed to evaluate the effectiveness of a family intervention in improving the family members' mental health and HRQoL. Intervention details have been published (5, 50). Parents were recruited in several German and Swiss inpatient psychiatric units based on the patients' and their families' availability and willingness to take part. Study participation required at least one formal ICD-10 mental diagnosis made by the clinicians in charge. Participants with acute symptomatology requiring inpatient treatment were excluded. Ethical approval was provided by the Ethics Committee of the regional Medical Association (Hamburg, Germany). All adult participants gave written informed consent. Written assents of children (<18 years) and their parents' permission were obtained.

### Participants

The overall project sample compromised of 216 families including *n* = 216 parents with mental illness, *n* = 145 partners and 338 children. Eight parents were excluded because they had more than 30% questionnaire related missing values. We considered only children for analyses that fulfilled the CBCL 4–18 required age range of 4–18 years. The final sample under analysis consisted of *n* = 208 parents that were formally diagnosed by clinicians with at least one mental ICD-10 diagnosis, and *n* = 197 children and adolescents aged 4 to 18 years.

### Measures

#### Parental Health-Related Quality of Life

The EQ-5D-3L (55, 56) measures current HRQoL. We assessed self-reported global HRQoL with the index value and with a visual analog scale (VAS). The index represents a single number that is calculated from individual health profiles using country specific value sets. Hence, the EQ5-D-3L index is based on a valuation of health states by the German general population and indicate the preferences of the reference population. The best possible health state is represented by an index score of 1, whereas death is valued with an index score 0. The VAS has values from 0 to 100 with higher scores suggesting greater health. Domain-specific HRQoL was indicated by five subscales (mobility, self-care, usual activity, discomfort, anxiety and depression). The subscales and the index are rated within three severity levels (1 = no symptoms, 2 = moderate symptoms, 3 = severe symptoms). For study purposes we summarized level 2 and 3 as “any problem.” The EQ-5D-3L is a moderately valid instrument to assess HRQoL in mentally ill adults (55). It has demonstrated good psychometric properties (55–57).

#### Parental Burden of Psychopathology

Parents with mental illness rated their perceived burden of mental illness on the Brief Symptom Inventory (BSI) (58). The questionnaire has 53-items that are answered on a five-point response scale (0 = not at all to 4 = extremely or 0 = never to 4 = always). We used the Global Severity Index (GSI) for analyses that indicates the subjective burden of current or past symptoms (somatization, obsessive-compulsive, interpersonal sensitivity, depression, anxiety, hostility, phobic anxiety, paranoid ideation, and psychoticism), as well as the number and intensity of symptoms. GSI scores range from 0 to 4 with higher scores indicating greater burden of mental illness. Good psychometric properties have been established for the BSI (58). In this context, the GSI had an excellent internal consistency (Cronbach's α = 0.96).

#### Parental Coping With Psychopathology

Patients self-reported their predominant coping behavior from 1 = not at all to 5 = very much on the Freiburg Questionnaire of Coping with Illness (FKV-LIS) (59). The questionnaire has 23 items that cover adaptive (active problem-oriented coping, distraction and self-growth) and maladaptive coping strategies (depressed processing style, religiosity and quest for meaning, trivialization and wishful thinking). According to the authors, the subscales' internal consistency ranged from Cronbach's α = 0.68 to 0.77 (59). The internal consistency in this study ranged from Cronbach's α = 0.52 (religiosity and quest for meaning) to 0.71 (active problem-oriented coping).

#### Family Functioning

Parents with mental illness rated their family functioning on the 40-item General Family Questionnaire (60). The overall family functioning was analyzed with the total score that is the sum of seven subscales (task fulfillment, role behavior, communication, emotionality, affectivity of relations, control, values and norms). Items are rated on a four-point response scale ranging from 0 = completely true to 3 = not true at all. The total score ranges from 0 to 120 with higher scores reflecting greater family dysfunction. The questionnaire possesses about good psychometric properties (60). The total score in this study had an excellent internal consistency of α = 0.94.

#### Social Support

The OSSS-3 (61) measures the patients' perceived social support with three items that ask respondents to self-report the number of close confidants, the sense of concern from other people, and the relationship with neighbors and the accessibility of practical help. The response scale of the first item has been adapted for study purposes (1= none, 2 = 1–2, 3 = 3–4, 4 = 5–6 and 5 = more than 6). The total score is calculated by summarizing those three items. It could range from 3 to 15 with higher scores indicating greater social support. Good validity and reliability was established for the OSSS-3 in a representative German sample with 2,524 participants (62). The modified version in this study demonstrated an internal consistency of α =0.31.

#### Psychopathology in Children

The parent-proxy version of the Child Behavior Checklist 4–18 (63, 64) rates maladaptive emotional and behavioral symptoms in children aged 4–18 years. Respondents answer 118 items on a three-point response scale from (0 = not at all to 2 = often). A total problem score was generated that covers both internalizing and externalizing behavior. The total score can range from 0 to 111. Higher scores reflect greater mental illness. The questionnaire has good psychometric properties (63, 64). Here, the total score had an excellent internal consistency (Cronbach's α = 0.95).

### Data Analysis

We first compared the observed probability of the occurrence of any problem in our sample with German population norms to examine whether our sample's global and domain-specific HRQoL (mobility, self-care, usual activities, pain/discomfort, anxiety/depression) differentiated significantly from the reference population (65). This was done with a one sample test of proportions for domain-specific HRQoL) and with a one sample *t*-test for global HRQoL. The impact of multiple factors (patients' physical and mental health, parental coping, family functioning, social support, child mental illness, parental age and gender) on parents' global HRQoL was evaluated with multiple linear regression analyses with backward elimination. Their impact on specific HRQoL was examined with binary logistic regression analyses. Whereas all factors are represented in Model 1, Model 2 includes factors with significance *p* < 0.100. As parents were able to make more than one proxy-rating for their children, child proxy-ratings were summarized for each family. Standardized coefficients, standard errors and statistical significance were calculated for each determinant, and overall model fit was reported. All variables were based on self-reported raw values. All continuous factors were mean-centered. Participants with more than 30% variable missing values were excluded. The remaining questionnaire-related missing values were imputed according to the Expectation-Maximization algorithm (EM) (66). Statistical significance was set at α ≤ 0.05 both one and two-sided. All statistical analyses were performed using IBM SPSS 25 (IBM, Armonk, NY, USA).

## Results

### Descriptive Statistics

Table 1 displays the characteristics of *n* = 208 parents with mental illness and *n* = 197 children and adolescents aged 4 to 18 years. Parents with mental illness had a mean age of M = 40.04 (SD = 7.15). 75.5% were female. The majority of parents had 10–13 years of school education. Participants were primarily diagnosed according to the ICD-10 with mood (affective) disorders (F30-F39), followed by disorders of adult personality and behavior (F60-F69), neurotic, stress-related and somatoform disorders (F40-F48), and other disorders. Almost half of the parents had comorbid mental disorders. The average parent with mental illness self-reported a remarkable burden of mental illness (BSI raw score ≥ 0.62). The participants' predominant coping behavior were active problem-oriented coping followed by a depressed processing style. Half of the parents self-reported suffering from physical impairments. Parents scored on average M = 36.76 (SD = 15.33) on family functioning, M = 9.41 (SD = 1.63) on social support. Patients reported moderate health-related quality of life. The majority of them expressed symptoms in anxiety/depression, pain/discomfort and usual activities. Less prevalent were symptoms with self-care and mobility. About half of the sample was married. Parents had on average two children with a mean age of M = 12.17 (SD =3.09). They proxy-reported for their children total CBCL-4-18 scores of M = 38.02 (SD = 25.47).

**Table 1:** Characteristics of parents with a mental illness.

**Characteristics**	***n* (%)**	**M (*SD*)**	**Possible range of score**
Sociodemographic data			
Age (in years)^1^		40.04 (7.15)	
Female^1^	157 (75.5%)		
Married^1^	112 (54.6%)		
School education^1^			
11–13 years education	64 (32.0%)		
10 years education	90 (45.0%)		
9 years education	42 (21.0%)		
No secondary education	4 (2.0%)		
Mental health			
Mental diagnosis (ICD-10)^2^, ^a^			
F10–F19	3 (1.4%)		
F20–F29	10 (4.8%)		
F30–F39	120 (57.7%)		
F40–F48	25 (12.0%)		
F60–F69	49 (23.6%)		
F90–F98	1 (0.5%)		
Comorbidity (ICD-10)^2^	84 (40.4%)		
Burden of mental illness (BSI, GSI)^1^, ^b^		1.35 (0.69)	0–4
Parental Coping (FKV-LIS)^1^, ^c^			
Adaptive coping		3.02 (0.60)	1–5
Maladaptive coping		4.18 (0.85)	1–5
Physical impairments (*ad-hoc* item)^1^	102 (50.5%)		
Family functioning (FB-A, total score)^1^, ^d^		36.76 15.33)	0–120
Social support (OSSS-3, total score)^1^, ^e^		9.41 (1.63)	3–15
Health-related quality of life^1^, ^f^			
(EQ-5D, proportions of respondents scoring any problem and total scores)^1^			
Mobility	48 (23.1%)		1–2
Self-care	55 (26.4%)		1–2
Usual activities	135 (64.9%)		1–2
Pain/discomfort	143 (68.8%)		1–2
Anxiety/depression	183 (88.0%)		1–2
VAS		54.22 (19.67)	0–100
Index-G		0.56 (0.22)	−0.21–1.00
**Children**			
Number of children^1^		1.91 (0.93)	
Age (in years)^1^		12.17 (3.09)	
Female^1^	109 (55.3%)		
Child mental illness (CBCL 4-18)^1^, ^g^		38.02 (25.47)	0–111

*n = 208 parents with mental illness, n = 197 children and adolescents. Questionnaire-related scores were based on raw data; for measures, see text (Methods)*.

1 *based on parent self- and proxy reports*.

2 *based on proxy-ratings by clinicians*.

a *International Classification of Diseases (ICD-10) codes: mental and behavioral disorders due to psychoactive substance use (F10–F19), schizophrenia, schizotypal, and delusional disorders (F20–F29), mood (affective) disorders (F30–F39), neurotic, stress-related and somatoform disorders (F40–F48), disorders of adult personality and behavior (F60–F69), behavioral and emotional disorders with onset usually occurring in childhood and adolescence (F90–F98);*

b *Brief Symptom Inventory (BSI);*

c *Freiburg Questionnaire of Coping with Illness (FKV-LIS);*

d *General Family Questionnaire (FB-A);*

e *Oslo Social Support Scale (OSSS-3);*

f *HRQoL (EQ-5D);*

g *Child Behavior Checklist 4–18 (CBCL 4-18)*.

### The Health-Related Quality of Life of Parents With Mental Illness Compared to the German General Population

Table 2 displays the self-reported HRQoL scores of *n* = 208 patients with mental disorders after age standardization. Our sample reported significantly lower global and specific HRQoL across all HRQoL domains (most *p* < 0.001) compared to the reference population (65). The difference in proportions of respondents scoring any problem was most prevalent in the domain anxiety and depression followed by usual activities and pain/discomfort, and less pronounced with regard to self-care and mobility.

**Table 2:** Self-reported EQ-5D scores after age standardization, mean EQ VAS, EQ Index and proportions (%) of respondents scoring any problem.

**EQ-5D**	**German population norms**	**Sample**	***p***
Mobility	17.2%	23.1% [17.5, 29.4]	0.032^*^
Self-care	3.1%	26.4% [20.6, 33.0]	<0.001^***^
Usual activities	10.5%	64.9% [58.0, 71.4]	<0.001^***^
Pain/ discomfort	27.8%	68.8% [62.0, 75.0]	<0.001^***^
Anxiety/depression	4.5%	88.0% [82.8, 92.1]	<0.001^***^
VAS	*M* = 77.2	*M* = 54.22 [51.45, 56.98]	<0.001^***^
Index	*M* = 0.90	*M* = 0.56 [0.53, 0.59]	<0.001^***^

*n = 208. Square brackets indicate the 95% confidence interval; for details see text (Methods)*.

* *p < 0.05;*

** *p < 0.01;*

*** *p < 0.001*.

### Risk and Protective Factors Associated With Health-Related Quality of Life of Parents With Mental Illness

Results of the EQ-5D index and EQ-5D VAS (Table 3) indicate that self-reported physical health and burden of mental illness, as well as adaptive coping are significant risk and protective factors associated with global HRQOL. The better participants received their physical and psychological health, and the more parents with mental illness employed adaptive coping strategies like active problem-oriented coping and distraction and self-growth, the higher they rated their overall HRQoL. Physical health, psychological symptom burden and adaptive coping strategies explained 40% of the variance in the EQ-5D index and 35% of the variance in the EQ-5D VAS. The amount of explained variance slightly increased when only factors with significance levels smaller than *p* < 0.100 were included. Maladaptive coping, family functioning, social support, child mental illness, age and gender did not contribute to the model.

**Table 3:** Risk and protective factors associated with global HRQoL in parents with mental illness.

	**EQ5D index**		**EQ5D VAS**	
**Model**	**1^a^**	**2^b^**	**1^a^**	**2^b^**
**Fixed effects**	*b*	*b*	*b*	*b*
Physical health^1^	−0.24***	−0.22***	−0.17**	−0.16**
Psychological symptom burden^2^	−0.56***	−0.54***	−0.46***	−0.47***
Parental Coping^3^				
Adaptive	0.09	0.10	0.22***	0.23***
Maladaptive	0.01		0.04	
Family functioning^4^	0.01		−0.01	
Social support^5^	0.04		0.06	
Child mental illness^6^	0.04		−0.02	
Age^1^	−0.08		0.06	
Female^1^	−0.10		0.01	
Age by gender^1^	0.15		−0.05	
**Model fit**				
Adjusted R^2^	0.39	0.40	0.33	0.35

*n = 200. b = standardized coefficients; measures:*

1 *ad-hoc items*,

2 *BSI GSI*

3 *FKV-LIS total score*,

4 *FB-A total score*,

5 *OSSS-3 total score*,

6 *CBCL 4–18 total score;*

a *Model 1 contains all factors*,

b Model 2 contains factors with p <0.100 identified with backward elimination; all continues factors were mean-centered; analyses were conducted with multiple linear regression analyses and were based on raw data; for details see text (Methods). *p <0.05; **p <0.01; ***p <0.001.

Supplementary Tables 1–3 display the determinants of domain-specific HRQoL. The significant factors associated with mobility were patients' self-reported physical health and child mental illness. Self-care was mainly associated with the patient's psychological symptom burden. Usual activities and pain/ discomfort were associated with both the patient's mental and physical health. Anxiety and depression were mainly associated with the patients' physical health and child mental illness. Across all domain-specific outcomes of HRQoL, parents with mental illness rated their HRQoL higher when they had few physical or mental complaints. In addition, child mental illness was associated with any mobility- and anxiety/discomfort-related symptoms. Risk and protective factors in final models explained 12% of variance in mobility 14% in self-care and usual activity, 19% in pain and discomfort, and 12% of variance in anxiety and depression. Parental coping, family functioning, social support, the patients' age and gender did not contribute to any of the final domain-specific models.

## Discussion

The main purpose of the present study was to analyze risk and protective factors associated with HRQoL in parents with mental illness. In line with previous research on various psychopathologies (10–12, 67), parents in our sample self-reported significantly lower global and specific HRQoL than the German reference population. The most pronounced dissatisfaction with HRQoL was reported for the aspects that relate to anxiety and depression followed by usual activities, pain and discomfort. Global HRQoL was primarily associated with self-reported physical and mental health, as well as adaptive coping behavior. The better the participants' physical and psychological condition, and the more parents used adaptive coping strategies like active problem-oriented coping and distraction and self-growth to deal with the challenges of their mental illness, the better they perceived their overall HRQoL. The results are consistent with previous research demonstrating the positive impact of adaptive coping on symptom severity, burden of mental illness, comorbidities and illness duration (31–34) and the relevance of physical (68, 69) and mental health (10–12, 67) for individual HRQoL. Against our expectations (36, 44, 45, 47–49), family functioning, social support, child mental illness and the age and gender of parents with mental illness did not appear to be relevant for the outcome. Future research may assess whether the irrelevance of those factors relate to the measurement instruments, the recruitment strategy or reflect actualities. It may also be possible that the EQ-5D failed to detect the actual effects of psychological constructs like family and social functioning, because it provides a very limited coverage of themes identified by individuals with mental health symptoms (70). Items and subscales of the EQ-5D focus more on physical rather than mental components. Results are mixed regarding its application in mental health settings (71–73), although it has demonstrated moderate validity in the assessment of HRQoL in adults with mental illness (55).

With regard to domain-specific aspects of HRQoL, the patients' satisfaction with mobility was significantly associated with physical health and child mental illness. The ability to move is crucial to conduct an independent life and it is an important aspect of physical quality of life (74). Independent of their physical condition, caregivers of children with mental illness may feel restricted in their mobility due to practical and emotional challenges associated with their children's mental illness (e.g. financial burden, marital stress). They may also have to alter their schedules to accommodate mental health appointments of their children. Problems with self-care were primarily associated with the patients' psychological symptom burden. This is consistent with other research (75). For example, one study showed depressive symptoms among diabetes patients were associated with less self-care behaviors such as adherence to exercise regimes and diet (75). Difficulties with usual activities, pain and discomfort were associated with the patients' mental and physical health. Results are in line with other studies involving participants whose physical (76) and mental illness (77, 78) interfered with their daily routine. In our case, physical health complaints may reflect somatic expressions of mental illness like pain, weakness, shortness of breath and discomfort (79). Any problems with anxiety and depression were primarily associated with a worse physical health and more mental health symptoms in children. This finding is in accordance with studies that have reported a high prevalence of mental health symptoms in physically disabled individuals (80, 81) and in parents who care for children with mental health symptoms (44, 45). A large amount of variance of global and domain-specific HRQoL remained unexplained. Future studies may focus on the identification of additional relevant risk and protective factors associated with HRQoL that were not be considered in this study.

### Limitations

This study had several limitations that should be taken into account when interpreting the results. Due to the simultaneous assessment of variables in this cross-sectional design, we cannot make inferences about the temporal relationship between dependent and independent variables. Longitudinal data analyses are needed for causal inferences. Another constraint is the limited number of risk and protective factors that have been considered for analyzing HRQoL in parents with mental illness. Although parents reported their perceived symptom burden, we did not differentiate between psychopathologies, symptom frequency, chronicity, prognosis, and illness duration. Similarly, we did neither define (e.g., by level of care) nor did we objectify physical complaints (e.g., by questionnaires, medical records). Consequently, we could not differentiate between medical impairments like a broken leg and somatic symptoms originating from mental illness. Future studies should also pay attention to the respondent's socioeconomic status, which was not assessed in the present study, but appears to be relevant irrespective of the presence of mental health symptoms. Furthermore, results may be slightly biased due to the recruitment strategy that may have attracted mainly families with higher family functioning, and due to the high proportion of females in our sample. Women generally report more problems in all EQ-5D dimensions across countries (65). Future research may also use the structured clinical interview for DSM-5 [SCID-5-RV, (82)] to document specific mental health symptoms and to ensure that the clinical inclusion criteria are met.

## Conclusion and Implications

Mental disorders have a substantial impact on global and specific aspects of HRQoL of parents with mental illness. The HRQoL assessment of patients with mental health symptoms is crucial for the evaluation of the perceived symptom burden and the (cost-) effectiveness of psychological treatments. The identification of modifiable risk and protective factors that are linked to the patients' HRQoL is important, as those can be targeted in clinical interventions to ultimately improve the patients' and their families' HRQoL. Consistent with previous research, our results indicate that the alleviation of mental health symptoms as well as physical impairments appear to be the most important factors in improving HRQoL. In addition, clinicians may promote adaptive coping behaviors in parents with mental illness and improve their children's mental health in order to increase the patients' HRQoL. Empirical research like the present study can establish new knowledge about the concept of quality of life, its relevance and components in populations with mental illness.

## Data Availability Statement

The raw data supporting the conclusions of this article will be made available by the authors, without undue reservation.

## Ethics Statement

The studies involving human participants were reviewed and approved by Ethics Committee of the regional Medical Association, Hamburg, Germany. Written informed consent to participate in this study was provided by the participants' legal guardian/next of kin.

## Author Contributions

AR was responsible for the conceptualization and formal analysis of the present research objectives, as well as for visualizing, writing, and editing the original draft. The latter was critically reviewed by MS, AD, MB, and SW-G. Data were provided by the CHIMPS study group with SW-G being the principal researcher. SW-G, RK, and ML were significantly involved in the conception of the CHIMPS project with methodological support provided by AD and KW. SW-G, BA, and KW coordinated the multicenter project (steering committee). SW-G, BA, SW, and RK were responsible for recruitment. BA and MB were responsible for data curation. All authors have read and agreed to the published version of the manuscript.

## Funding

This research was funded by the German Federal Ministry of Education and Research (BMBF), Grant Number 01GY1337.

## Conflict of Interest

The authors declare that the research was conducted in the absence of any commercial or financial relationships that could be construed as a potential conflict of interest.

## Publisher's Note

All claims expressed in this article are solely those of the authors and do not necessarily represent those of their affiliated organizations, or those of the publisher, the editors and the reviewers. Any product that may be evaluated in this article, or claim that may be made by its manufacturer, is not guaranteed or endorsed by the publisher.
