# Supplementary material for: Risk and Protective Factors Associated With Health-Related Quality of Life of Parents With Mental Illness
Source: Front Psychiatry. 2021 Dec 1;12:779391. doi: 10.3389/fpsyt.2021.779391 (PMC8672802; doi:10.3389/fpsyt.2021.779391)
Supplement: Supplementary file 1 [file Table_1.docx]

**Supplementary Table 1**

*Risk and protective factors associated with mobility in parents with mental illness*

|  | Mobility | | | | |
| --- | --- | --- | --- | --- | --- |
| Model | 1 | |  | 2 | |
| Fixed Effects | *B* | Exp(B) |  | *B* | Exp(B) |
| Intercept | -0.36* |  |  | -0.70** | 0.50 |
| Physical health^1^ | -1.40** | 0.25 |  | -1.45*** | 0.24 |
| Psychopahtology^2^ | 0.61 | 1.84 |  |  |  |
| Parental Coping^3^ |  |  |  |  |  |
| Adaptive | -0.08 | 0.92 |  |  |  |
| Maladaptive | -0.51 | 0.60 |  |  |  |
| Family functioning^4^ | -0.01 | 0.99 |  |  |  |
| Social support^5^ | -0.09 | 0.92 |  |  |  |
| Child mental illness^6^ | 0.02* | 1.02 |  | 0.02** | 1.02 |
| Age ^1^ | -0.06 | 0.94 |  |  |  |
| Female^1^ | -0.57 | 0.56 |  |  |  |
| Age by gender^1^ | 0.05 | 1.05 |  |  |  |
| Model Fit |  |  |  |  |  |
| R^2^ (Cox&Snell) | .15 | |  | .12 | |

*Note*. *n* = 200. *B* = unstandardized coefficients Measures: ^1^ ad-hoc items, ^2^ BSI GSI ^3^ FKV-LIS total score, ^4^ FB-A total score, ^5^ OSSS-3 total score, ^6^ CBCL 4-18 total score; all continues factors were mean-centered; analyses were conducted with binary logistic regression analyses and were based on raw data; for details, see text (Methods). **p* < .05; ***p* < .01; ****p* < .001
