# Supplementary material for: Risk and Protective Factors Associated With Health-Related Quality of Life of Parents With Mental Illness
Source: Front Psychiatry. 2021 Dec 1;12:779391. doi: 10.3389/fpsyt.2021.779391 (PMC8672802; doi:10.3389/fpsyt.2021.779391)
Supplement: Supplementary file 2 [file Table_2.docx]

**Supplementary Table 2**

*Risk and protective factors associated with self-care and usual activity in parents with mental illness*

|  |  | Self-Care | | | | |  | Usual Activity | | | | |
| --- | --- | --- | --- | --- | --- | --- | --- | --- | --- | --- | --- | --- |
| Model |  | 1 | |  | 2 | |  | 1 | |  | 2 | |
| Fixed Effects |  | *B* | Exp(B) |  | *B* | Exp(B) |  | *B* | Exp(B) |  | *B* | Exp(B) |
| Intercept |  | -0.18 | 0.84 |  | -0.83*** | 0.46 |  | 1.51** | 4.51 |  | 1.14*** | 3.11 |
| Physical health^1^ |  | -0.77* | 0.46 |  | -0.57 | 0.50 |  | -0.75* | 0.47 |  | -0.67* | 0.51 |
| Psychopahtology^2^ |  | 0.86** | 2.37 |  | 0.91** | 2.42 |  | 1.18** | 3.24 |  | 1.12*** | 3.07 |
| Parental Coping^3^ |  |  |  |  |  |  |  |  |  |  |  |  |
| Adaptive |  | -0.06 | 0.95 |  |  |  |  | -0.38 | 0.68 |  |  |  |
| Maladaptive |  | -0.23 | 0.80 |  |  |  |  | -0.29 | 0.75 |  |  |  |
| Family functioning^4^ |  | 0.02 | 1.02 |  | 0.02 | 1.02 |  | -0.03 | 1.01 |  |  |  |
| Social support^5^ |  | -0.14 | 0.87 |  |  |  |  | 0.00 | 1.00 |  |  |  |
| Child mental illness^6^ |  | 0.01 | 1.01 |  |  |  |  | 0.00 | 1.00 |  |  |  |
| Age ^1^ |  | 0.10 | 1.01 |  |  |  |  | 0.06 | 1.06 |  |  |  |
| Female^1^ |  | -0.75 | 0.47 |  |  |  |  | -0.32 | 0.73 |  |  |  |
| Age by gender^1^ |  | -0.06 | 0.95 |  |  |  |  | -0.05 | 0.95 |  |  |  |
| Model Fit |  |  |  |  |  |  |  |  |  |  |  |  |
| R^2^ (Cox&Snell) |  | .16 | |  | .14 | |  | .16 | |  | .14 | |

*Note*. *n* = 200. *B* = unstandardized coefficients Measures: ^1^ ad-hoc items, ^2^ BSI GSI ^3^ FKV-LIS total score, ^4^ FB-A total score, ^5^ OSSS-3 total score, ^6^ CBCL 4-18 total score; all continues factors were mean-centered; analyses were conducted with binary logistic regression analyses and were based on raw data; for details, see text (Methods). **p* < .05; ***p* < .01; ****p* < .001
